# Test yourself

**Published:** 2013

Test your understanding of the concepts covered in this issue and discuss any points of interest with your manager or a colleague. *Produced in collaboration with the International Council of Ophthalmology (ICO)*.

**Table:** 

**1.**	**Think about ‘balancing the books’ and sustainability**	**True**	**False**
a	External donor funds are best used for training and capacity building	□	□
b	Governments are not responsible for paying for eye care	□	□
c	If you charge for services, everyone should pay the same	□	□
d	Pharmacy and spectacle sales are two areas where income can be generated	□	□
**2.**	**Think about patient flow, accounting and procurement**	**True**	**False**
a	Buying smaller quantities of consumables, more frequently, saves money	□	□
b	Intraocular lenses (IOLs) should be on the procurement list if a hospital offers cataract surgery	□	□
c	You don't need to have a computer to set up an accounting system	□	□
d	Reducing the number of times a patient must visit the hospital, e.g. for cataract surgery, saves costs	□	□

## ANSWERS

**1a. True. b. False.** Governments must cover some of the costs or organise health insurance, as in Ghana. **c. False.** Some patients will pay more for value-added services. **d. True.**
**2a. False.** Bulk buy medicines and consumables yearly or quarterly. **b. True. c.** True. You can start on paper. **d. True**.

